# Mental capacity and borderline personality disorder

**DOI:** 10.1192/pb.bp.115.052753

**Published:** 2017-02

**Authors:** Karyn Ayre, Gareth S. Owen, Paul Moran

**Affiliations:** 1 South London and Maudsley NHS Foundation Trust, London; 2 Institute of Psychiatry, Psychology and Neuroscience, King's College London; 3 University of Bristol

## Abstract

The use of the Mental Capacity Act 2005 in assessing decision-making capacity in patients with borderline personality disorder (BPD) is inconsistent. We believe this may stem from persisting confusion regarding the nosological status of personality disorder and also a failure to recognise the fact that emotional dysregulation and characteristic psychodynamic abnormalities may cause substantial difficulties in using and weighing information. Clearer consensus on these issues is required in order to provide consistent patient care and reduce uncertainty for clinicians in what are often emergency and high-stakes clinical scenarios.

Borderline personality disorder (BPD) is a severe mental disorder affecting around 1% of the population.^1^ It is associated with significant psychiatric comorbidity,^2^ impairment in social function^3^ and a high rate of service utilisation.^4^ Personality disorder as a whole is associated with reduced life expectancy.^5^ People with BPD may engage in self-harming behaviour as a way of regulating negative affect, particularly at times of crises.^6^ Assessing decision-making capacity in a patient with BPD who requires medical treatment following an act of self-harm is particularly challenging.^7^ In the overlap between the Mental Health Act 1983 and Mental Capacity Act 2005 (the Act), it is the decisions around physical healthcare treatment without consent, i.e. those that the Mental Health Act cannot be used to enforce unless treatment is recognised as treatment for mental disorder, that in our experience generate considerable anxiety. This is particularly true in a general hospital setting as exemplified in the tragic case of Kerrie Wooltorton.^8^

The Mental Capacity Act is the legal framework used in England and Wales for assessing capacity. It also provides protection to a clinician who makes decisions on behalf of an incapacitous patient, providing its terms are satisfied and the clinician is acting in the patient's best interests. Assessment of capacity is time- and decision-specific, however, in our clinical experience there is inconsistency surrounding the use of the Act with BPD patients. This is partly the result of disputes relating to the nosological status of personality disorder as a whole.^7,9^ Such disputes are discordant with increasing knowledge about the natural history of BPD and its neurobiological abnormalities and treatability. In this article we explore the key reasons for inconsistency on whether BPD has the potential to give rise to the ‘impairment of, or disturbance in the functioning of, the mind or brain’ criterion of the Act, as well as highlighting the need for clearer guidance on the use of the Act with such individuals.

## Applicability of the Act in borderline personality disorder

As indicated by Section 2(1) of the Act, patients must demonstrate an ‘impairment of, or disturbance in the functioning of, the mind or brain’ for the Act to apply – sometimes known as the ‘diagnostic threshold’. Applicability to patients with a psychotic illness or a severe affective disorder is rarely questioned, presumably because these conditions are clearly described and widely accepted as constituting mental disorders that have the potential to impair or disturb function.^7^ However, in one of the few discussions of the impact of personality disorder on competence to refuse treatment, Winburn & Mullen^10^ point out that personality disorder, although classed as such under the Mental Health Act 1983, ‘has always been considered to be at the margins of what constitutes mental disorder’. Although it is by no means an exhaustive list, paragraph 4.12 of the Mental Capacity Act Code of Practice^11^ does not include personality disorder among the other conditions it recommends as having the potential to cause impairment or disturbance in the functioning of the mind or brain. Indeed, as recently as the past decade, lingering doubts about the nosological status of personality disorders in general were still being voiced by senior figures in academic psychiatry.^9^

Over the past 10–15 years, the empirical evidence base for personality disorders in general and particularly for BPD has expanded substantially. Robust longitudinal studies have charted the natural history of BPD^12^ and shown that while symptomatic improvement is common, enduring impairment in social functioning^3^ is a defining feature of the condition. Cost of illness studies have shown that the costs of managing people with BPD exceed the costs associated with managing people with depression.^4^ Other studies have shown that the life expectancy of people with personality disorder is markedly reduced and that the loss of lived years is at least equivalent to that seen in schizophrenia.^5^ There is also growing evidence of underlying neurobiological abnormalities associated with BPD.^13,14^ Finally, the increasing number of well-conducted clinical trials which show that effective treatment is possible provides further evidence supporting the validation of the construct of BPD.^15^

In summary, as a result of considerable research endeavour, we now know that BPD is, without doubt, a valid category of mental disorder. As such, it must be considered ‘an impairment of, or a disturbance in, the functioning of the mind or brain’. Therefore, not only is the Mental Capacity Act framework applicable to people with BPD, but it is clinically inappropriate not to consider its relevance when assessing people with BPD. There is a need for greater consensus regarding this, to eradicate the assumption that capacity assessments, *tout court*, cannot apply to such patients.^7,11^

Perhaps another reason why this assumption has been so pervasive relates to Parsons's notion of the ‘sick role’.^16^ This illustrates the link between illness and its social benefits: among these, the absolution of responsibility.^17^ The doctor's role is key, as it is the doctor who confers this role on the patient and provides social sanction for receipt of those benefits.^16^ Doctors are often divided as to whether patients with BPD can be thought of as being ill and thus entitled to a sick role.^9,18^ As the Act requires the presence of an ‘impairment’ or ‘disturbance in function’ to be applicable, one might view its application to BPD patients as being synonymous with sanctioning an adoption of the sick role. Such a view may not sit comfortably with a clinician who may view a volitional act of self-harm as being ‘manipulative’.^9,18^

The issue of volitional control and, by inference, manipulation, therefore seems the crux of the matter. Pickard^19^ points out that it is hard to accept that patients with a personality disorder completely lack control over their actions. She qualifies this by pointing out they ‘may not always have full conscious knowledge of why they are behaving as they do’.^19^ Similarly, Szmukler has speculated that suicidal impulses may sometimes stem from ‘an inner disturbance the [person] finds difficult to describe’.^7^ The question for clinicians is that, in situations where high-stakes decisions must be made, how does the phenomenology of BPD impair an individual's ability to understand and reflect on both the risks and benefits of treatment, and also the motivation underlying their behaviour?

## How does BPD affect capacity?

Fuchs^20^ identified that, at the extremes of emotional dysregulation, BPD patients become enveloped in that mental state to the extent that they are unable to view things objectively. Over time, repetition of this cycle leads to the chronic feelings of emptiness that characterise the disorder, meaning that patients ‘miss the experience of agency or authorship of their life’.^20^ Broadly speaking, this key concept can be illustrated in two general clinical scenarios.

The first is a patient presenting as an emergency at the extreme of an episode of emotional dysregulation. Self-harming behaviours may serve an affect regulation function^6^ and assessment of capacity to accept or refuse treatment following a severe episode of self-harm is a common clinical scenario. The framework for decision-making, per the Act, requires the patient to understand the pros and cons of treatment for their condition. If the self-harm is life threatening, accepting treatment would therefore be life saving, and to refuse, by inference, a decision likely to result in death.

To ‘use or weigh’ relevant information about options in the process of deciding is the element of the capacity test that causes most interpretative difficulty in BPD. As Principle 4 of the Act states: ‘a person is not to be treated as unable to make a decision merely because he makes an unwise decision’. Deciding to refuse life-saving treatment may be unwise, but it is not the decision *per se* that we are assessing, rather how ‘accountable’ the patient is for the decision.^21,22^ Elliot has argued that in depression, even though patients may understand the risks, ultimately the disorder may affect whether they ‘care’ about that risk, thus reducing the ‘authenticity’ of the decision.^22^ If depression can lead to a pathological lack of ‘care’ about one's own interests, could the drive to emotionally regulate via self-harm lead to a pathological ‘resistance’ to acting in one's own best interests that robs BPD patients of decision authenticity?

Research has suggested that key interpersonal schemas in BPD include sadomasochistic behaviour, where patients hurt themselves in an internalised ‘punitive parent’ mode.^23^ The relevance to treatment refusal here seems clear. In addition, as Szmukler points out, any capacity assessment is essentially a dynamic between the patient and the doctor.^7^ Refusal of the doctor's recommendation could also be viewed within this sadomasochistic paradigm. This is surely the source of the sense of ‘manipulation’^18^ frequently felt by doctors treating these patients.

The great trap in these scenarios is assuming that refusal of life-saving treatment is equivalent to a wish to die and an acceptable ‘unwise’ decision. While this may indeed be the case in some instances, this *de facto* assumption endangers the lives of some BPD patients, as in some cases the decision to refuse *per se* may simply be a manifestation of the disorder, rather than a carefully considered wish to die. The risk to these patients is compounded by an intolerance of ‘manipulation’ felt by the doctor, who in turn may take this as evidence that the patient has full insight into the situation and accept their refusal as an unwise but capacitous decision. In summary, as a direct consequence of the mental disorder itself, BPD patients may unwittingly become caught up in a destructive iatrogenic cycle of harm.

A second clinical example which aptly illustrates the lack of ‘authorship’ of life^19^ that may occur for people with BPD while dealing with doctors has been provided by Winburn & Mullen.^10^ They describe the case of a BPD patient who was judged incapacitous to refuse a potentially life-saving blood transfusion. Her decision to refuse treatment was viewed as a consistent, chronic behavioural pattern and overall constituted a ‘disturbed form of engagement … rather than an effort to disengage’. Case law reflects these views, as seen in the case of *B v Croydon Health Authority*,^24^ where a young woman with BPD was starving herself to the point where enforced nasogastric feeding was considered. Lord Justice Hoffman wrote in his judgment that he found it difficult to conclude that the patient had capacity, despite her seeming to have a good understanding of the risks and options. It was this that made him question whether her choice was truly autonomous, because, while being able to make cogent and articulate statements about her wishes, it was hard for him to deem someone capacitous when she is ‘crying inside for help but unable to break out of the routine of punishing herself’.

## How this affects clinical practice

The assessment of mental capacity in BPD patients is complex and may therefore cause clinicians significant anxiety where high-stakes decisions are to be made. It is conceivable that such anxiety may lead to risk-averse practices. In her review of suicide risk management in BPD patients, Goodman^25^ highlighted the influence of medico-legal concerns on clinicians, by referring to a survey^26^ that had shown that 85% of clinicians working with BPD patients had, within the past year, practised in a way ‘that would relieve their anxiety over medicolegal risks’. In our example of the BPD patient refusing life-saving treatment following self-harm and where capacity is marginal, risk-averse practice would presumably involve erring on the side least likely to result in death, i.e. a judgement of incapacity, detention and enforced treatment.

However, Pickard^19^ points out that it is particularly in the interests of patients with BPD that we attribute decision-making responsibility to them where possible, as this is the basis of some of the most effective psychological treatments for BPD, where self-control and mentalisation development are key. Szmukler suggests that when capacity could be argued from both sides, ‘one might conclude that … the patient's account, although not the one preferred by the clinician, is an adequate one, and sufficient to demonstrate that the patient has capacity’.^7^ Law states that ‘with regard to the degree of incapacity the nearer to the borderline the more weight must in principle be attached to [the patient's] wishes and feelings’.^27^ In application to BPD this would appear to imply that if the incapacity is only marginal the patient should, in effect, be approached as if with capacity.

So how do we balance over- and under-attributing capacity to BPD patients in clinical practice? Buchanan's work^28^ is relevant to this problem. He describes that when capacity is in doubt, we may vary our threshold for deciding what constitutes true incapacity, based on the stakes of the decision. Thus, when the negative consequences of a decision are likely to be severe, the clinician would require a more robust demonstration of capacity.^28^ In essence, the clinician is balancing possible infringement of autonomy with negative consequences of the outcome of the decision. Ultimately, capacity is judged legally to be either present or absent, but as Lord Donaldson pointed out in the case of *Re T (Adult: Refusal of Treatment)*,^29^ doctors should consider whether the capacity that is there is ‘commensurate’ with the seriousness of the decision.

One might argue that proportionality merely reflects the clinicians' increasing anxiety about higher-stakes situations, thus not addressing the underlying problem: that there is little consensus and guidance on whether and how BPD may affect decision-making. Clearer guidance and consensus on how BPD may affect decision-making abilities in different clinical scenarios will reduce anxiety for clinicians and may help the Act become more predictable in its application.

## Conclusions

Borderline personality disorder is a mental disorder. The use of the law in treating patients with BPD should be predictable and its application to clinical scenarios reproducible. The current use of the Mental Capacity Act 2005 in assessing decision-making capacity in such patients is lacking in these respects.

While BPD should be viewed as a mental disorder, this only means the Act is applicable; it is not synonymous with the view that people with BPD necessarily lack capacity for decision-making or responsibility for their actions. The psychopathology of BPD and specifically the way this affects the ‘using and weighing’ element of decision-making capacity is extremely complex and not acknowledged widely enough either in clinical practice or within the Mental Capacity Act itself. This leads to inconsistency in patient care. Further research into this field, along with clearer clinical consensus and legal guidance, is urgently required.
